# Open, prospective, randomised, multicentre trial on quality assessment of declined liver grafts by normothermic ex situ machine perfusion for decreasing time to transplantation (ExTra trial): a study protocol

**DOI:** 10.1136/bmjopen-2025-114833

**Published:** 2026-08-13

**Authors:** Simon Moosburner, Joseph M G V Gassner, Igor M Sauer, Mateja Mandac, Dominik P Modest, Dario Zocholl, Regina Stegherr, Madhukar S Patel, Julia Wendon, Christoph Roderburg, Markus Selzner, Andreas Pascher, Uli Fehrenbach, Johann Pratschke, Nathanael Raschzok

**Affiliations:** 1 Department of Surgery, Campus Charité Mitte | Campus Virchow-Klinikum, Charité - Universitätsmedizin Berlin, corporate member of Freie Universität Berlin and Humboldt-Universität zu Berlin, Berlin, Germany; 2 BIH Biomedical Innovation Academy, BIH Charité Clinician Scientist Program, Berlin Institute of Health at Charité– Universitätsmedizin Berlin, Berlin, Germany; 3 Department of Hematology, Oncology and Cancer Immunology, Charite - Universitatsmedizin Berlin, corporate member of Freie Universität Berlin and Humboldt-Universität zu Berlin, Berlin, Germany; 4 Institute of Biometry and Clinical Epidemiology, Charité - Universitatsmedizin Berlin, corporate member of Freie Universität Berlin and Humboldt-Universität zu Berlin, Berlin, Germany; 5 Department of Surgery, Division of Surgical Transplantation, The University of Texas Southwestern Medical Center, Dallas, Texas, USA; 6 Institute of Liver Studies, School of Immunology and Microbial Sciences, King’s College Hospital, London, UK; 7 Department of Gastroenterology, Hepatology and Infectious Diseases, University Hospital of Düsseldorf, Düsseldorf, Germany; 8 Department of Surgery, Ajmera Transplant Centre, University Health Network, Toronto General Hospital, Toronto, Ontario, Canada; 9 Department of General, Visceral and Transplant Surgery, University Hospital Münster, Münster, Germany; 10 Department of Radiology, Charite - Universitatsmedizin Berlin, corporate member of Freie Universität Berlin and Humboldt-Universität zu Berlin, Berlin, Germany

**Keywords:** Randomized Controlled Trial, SURGERY, TRANSPLANT MEDICINE

## Abstract

**Introduction:**

Liver transplantation remains the only curative option for end-stage liver disease, yet high waitlist mortality and organ scarcity continue to challenge the field. The randomised multicentre ExTra trial aims to decrease time-to-transplant by safely improving access to transplantation through viability assessment of otherwise discarded grafts with normothermic machine perfusion (NMP).

**Methods and analysis:**

The ExTra trial is a prospective, randomised, multicentre study conducted across German transplant centres. Participants listed for liver transplantation with a reMELD-Na-Score ≤21 (refitted MELD-Natrium Score) who are not eligible for Standard or Non-Standard Exceptions will be randomised for a 1-year intervention period into two groups: the experimental arm, in which they have—in addition to regular organ allocation—the extra option to receive a graft that was initially deemed non-transplantable based on clinical criteria, but meets specified quality criteria after at least 4 hours of NMP, and the control arm, which consists of conventional allocation procedures alone. The primary endpoint is time-to-transplant, defined as the period between randomisation and transplantation. Secondary outcomes include competing events (death, disease progression or recovery without transplantation), graft and patient survival, quality of life and cost-effectiveness. Additional analyses will assess early graft function, length of hospital stay, biliary complications and 1-year post-transplant outcomes. A centralised biobank will collect perfusate, tissue and blood samples for biomarker research. Safety will be monitored through quarterly reviews by a Data Safety Monitoring Board.

**Ethics and dissemination:**

The study protocol received approval from the Institutional Review Board of the Charité – Universitätsmedizin Berlin on 2 April 2025 (EA1/04/25). Participating study centres will need local Institutional Review Board approval prior to initiation. This study complies with Good Clinical Practice guidelines and all applicable national and local regulations. Results will be disseminated during international and national conference meetings and through publication in a peer-reviewed journal with open access.

**Trial registration number:**

NCT06874296.

**STRENGTHS AND LIMITATIONS OF THIS STUDY**

- Prospective, randomised, multicentre study design evaluating back-to-base normothermic machine perfusion (NMP) for declined liver grafts.
- Predefined viability criteria, ensuring reproducible organ assessment and minimising intercentre variability.
- Comprehensive endpoint framework combining waitlist period, perioperative outcomes and graft and patient survival, enabling a clinically meaningful evaluation of NMP effectiveness in expanding the donor pool.
- Multicentre study design increases methodological robustness and reduces the risk of centre-specific biases, although practice differences in postoperative management may still influence outcome variability.
- Reliance on the real-world availability of declined donor grafts that fulfil predefined NMP viability criteria may impede reaching the planned number of primary endpoint events.

## Introduction

### Background and rationale

 Liver transplantation (LT) is the preferred treatment for patients with advanced liver cirrhosis, hepatocellular carcinoma within Milan criteria and severe metabolic or autoimmune liver disease.^1^ However, the number of patients on the liver transplant waiting list exceeds the number of available organs. In Germany, the success of LT has been undermined by a dramatic decline in organ donations over the past decade.^2 3^ In 2022, only 748 out of 1296 patients (58%) on the waiting list received a liver graft.^4^ The outcome for patients on the waiting list remains unsatisfactory: A study from Essen showed that the 12-month survival rate without transplantation was 46%, compared with 78% after a liver transplant.^5^ Comprehensive data from the Eurotransplant registry show that between 2006 and 2015, 32% of patients were removed from the waiting list because they became too ill to receive a graft or died while waiting.^6^ This problem particularly affects patients with a lab MELD ≤25 (Model for End-Stage Liver Disease) who are not eligible for Standard or Non-Standard Exception criteria. Patients whose disease severity is not adequately reflected by lab MELD can, at the initiative of the transplant centre, be requested for an exceptional MELD; disease and country-specific rules apply.^7^ These patients wait significantly longer for a graft (2022: 112 vs 54 days, p<0.001) and have a significantly lower 12-month transplantation rate (44% vs 70%, p<0.001, unpublished data from Eurotransplant) compared with the general population of listed patients. The imbalance between supply and demand is further exacerbated by the fact that about 24% of all liver grafts offered for transplantation in Germany are declined due to the donor’s age or overall morbidity.^8^ This issue exists worldwide, with discard rates in the USA being similarly high (13%).^9^

The liver allocation process in Germany is strictly regulated and managed by Eurotransplant, in cooperation with the German organ procurement organisation ‘Deutsche Stiftung Organtransplantation’ (DSO, German Organ Transplantation Foundation). Each organ is allocated according to a specific algorithm. The decision to accept or reject a liver graft for a recipient is based on appropriately matching the donor’s clinical data with that of a potential recipient, as well as logistical considerations. To address the current organ shortage, liver grafts from donors with extended criteria (ECD) are increasingly used for transplantation.^10^ However, up to 24% of all liver grafts offered for transplantation are declined, mainly due to the donor’s age, macrosteatosis or long cold ischaemia time, as these factors are associated with an increased risk of graft dysfunction, graft failure and death, particularly in recipients with a high MELD score.^8 11–13^ The decision-making process is primarily based on the subjective expertise of the accepting transplant surgeon, supported by scores that consider both donor and recipient factors.^14 15^ Objective tools for assessing graft function are not routinely used in clinical practice for conventional static cold storage. For hypothermic oxygenated liver machine perfusion (HOPE), there are reports for measuring flavin mononucleotide and nicotinamide adenine dinucleotide to assess graft quality, although there is still ongoing need for prospective trials to internationally validate these criteria.^16 17^ Pilot studies have shown that normothermic machine perfusion (NMP) enables the assessment of organs clinically deemed unsuitable for transplantation using viability criteria such as lactate clearance or bile production.^18 19^

Transplantation of initially declined liver grafts following quality assessment by NMP has been investigated in eight non-randomised, single-centre clinical studies in the UK, the Netherlands, Australia, Italy, and the USA.^20–26^ The commercially available OrganOx Metra (OrganOx, Oxford, UK), the Liver Assist device (XVIVO B.V., Groningen, Netherlands), the Aferetica PerLife (Aferetica s.r.l., Bologna, Italy) and a non-commercial device developed in Cleveland were used in these studies. The OrganOx Metra and the institutionally developed device were used for pure end-ischaemic perfusion,^20 21 23 24 26^ while the protocol established in Groningen for the Liver Assist device consisted of 1 hour of HOPE, followed by controlled oxygenated rewarming (COR) and 2.5 hours of NMP for quality assessment.^22 25^ This protocol was also used in a modified form (90 min of COR and dual HOPE) in one study that employed both the Liver Assist and Aferetica PerLife devices.^27^

The ExTra trial will randomise patients on the waiting list to demonstrate that declined liver grafts, which meet predefined quality criteria after at least 4 hours of NMP, can be used to reduce the waiting time for LT in patients with a low MELD score who are not eligible for Standard or Non-Standard Exceptions. This is of clinical relevance given the increasing number of livers declined for transplantation in Germany, despite new technologies enabling quality assessment of these potentially transplantable organs. The utilisation rate of grafts, safety, cost-effectiveness and the effect on the waiting list practice will be evaluated as secondary endpoints. The results of this study could therefore change clinical practice in Germany and help address the medical-social dilemma of organ shortages in LT by integrating graft quality assessment through NMP into clinical practice. This could enable the acceptance of livers that are currently declined, which would certainly increase organ utilisation in Germany. To further leverage the impact of this clinical study, a translational companion study will be conducted to identify novel biomarkers for assessing and predicting graft quality in NMP.

### Objective

The objective of the ExTra trial is to demonstrate that the waiting time for LT in patients with a reMELD-Na score ≤21 (corresponds to labMELD ≤25), who are not eligible for Standard or Non-Standard Exception criteria, can be significantly reduced by using liver grafts that were initially deemed non-transplantable based on clinical criteria but meet predefined quality criteria after at least 4 hours of end-ischaemic NMP, compared with patients who are not offered this option. By analysing this procedure across participating study centres, we aim to safely transform organ acceptance practices in Germany, provided the results are successful.

## Methods and analysis

### Study design and timeline

**Figure 1:**
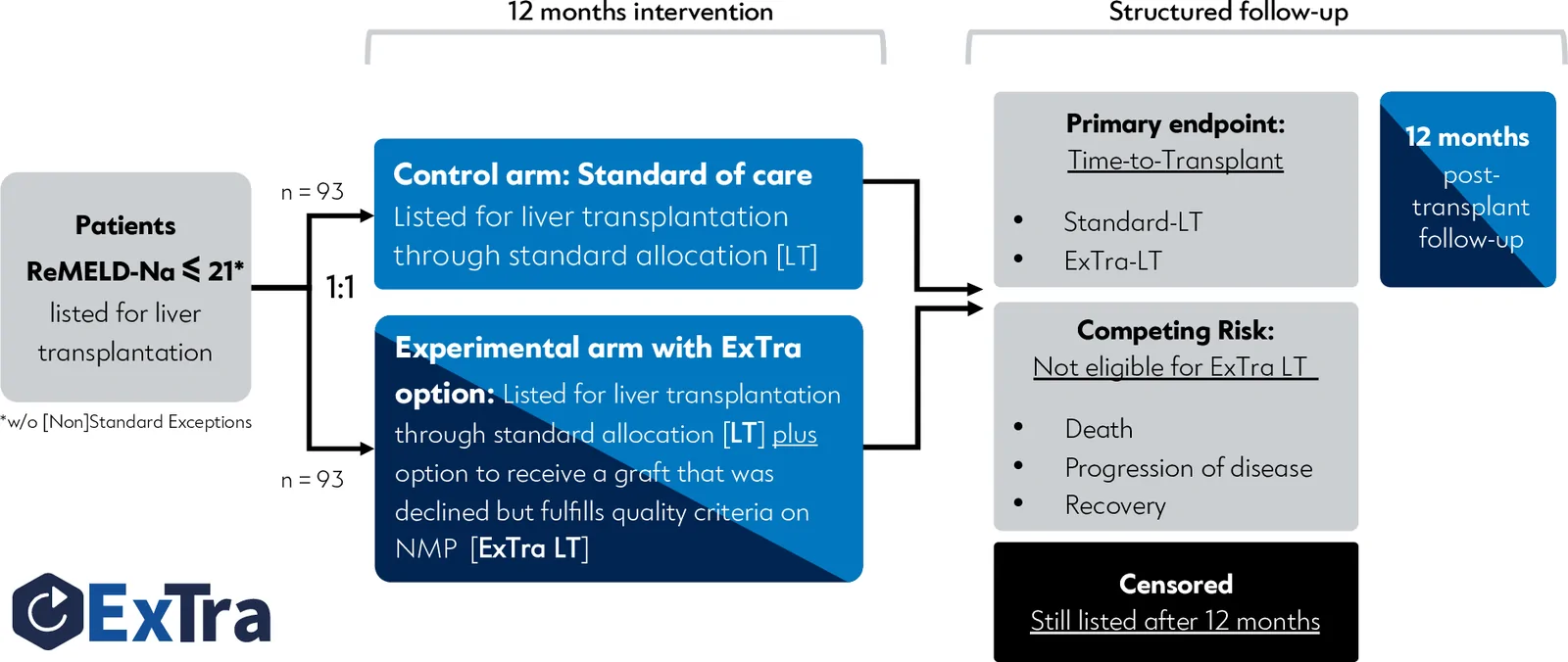
Visual presentation of patient timeline after randomisation in the ExTra trial. LT, liver transplantation; NMP, normothermic machine perfusion; reMELD-Na, refitted MELD-Natrium Score.

This is an investigator-initiated prospective, multicentre, randomised, open-label, two-arm clinical study. In total 186 patients will be randomised 1:1 to the experimental or control arm (figure 1). LT in the context of regular organ allocation can be performed in both arms according to the centre’s decision, that is, using static cold storage, hypothermic or NMP for organ preservation. Quality assessment of grafts judged clinically unacceptable by NMP is reserved for the experimental arm.

The proposed study duration is 66 months (1 June 2025–31 December 2030). The study started recruiting on 2 June 2025, with an anticipated recruitment period of 36 months, concluding on 31 May 2028. Each participant will be enrolled in the study for a maximum duration of 24 months, comprising an intervention phase of up to 12 months, followed by a 12-month structured follow-up period after transplantation (online supplemental table 1). The final participant follow-up is expected to be completed by 31 May 2030, after which the study will proceed with data analysis and dissemination activities. The study is scheduled to conclude by 31 December 2030.

### Eligibility criteria

All patients with a reMELD-Na ≤21 without eligibility for Standard or Non-Standard Exceptions, who are listed in the status transplantable for LT, and medically suitable and informed for transplantation with so-called marginal organs from donors, who meet extended donor criteria, will be recruited for the ExTra trial at the respective study centres.

#### Inclusion criteria

Before inclusion in the study, each patient is informed by the local study physician in charge about the nature, objectives, expected benefits and possible risks of the study, both orally and in writing. Patients are included while active on the waitlist. The duration of waitlist time before study inclusion is included in the randomisation stratification. Potential participants must demonstrate the capacity to (1) understand the study information, (2) retain the information, (3) weigh the risks and benefits and (4) communicate a clear decision. Only individuals who meet all four criteria are invited to participate. The decision is made by the treating physician.

The inclusion criteria are:

- Able to consent.
- ≥18 years old.
- Listed as status ‘transplantable’ by the transplant conference of the study centre for LT, according to the guidelines of the German Medical Association valid at the time of inclusion.
- reMELD-Na ≤21, not eligible for Standard or Non-Standard Exceptions.
  - Standard and Non-Standard Exceptions are defined in the Eurotransplant liver allocation manual.
- Medically suitable and informed for transplantation with an organ that fulfils extended donor criteria (Eurotransplant ECD criteria).
- Patient information and written consent to participate in the ExTra trial
  - If the patient can give consent but is unable to sign personally, a witness must confirm the oral explanation by signing.
- No participation in another interventional study during participation.

#### Exclusion criteria

- Listed for retransplantation.
- High-urgency listing.
- Listed for combined organ transplantation.
- Pregnancy.

### Recruitment

The primary recruitment measure should be a personal discussion as part of the information on LT during consultation hours. For this purpose, the respective doctors providing information and the transplant office in the recruiting centres will be informed and sensitised. In addition, information flyers will be made available in the respective transplant outpatient clinics so that potential study participants can inform themselves independently. All patients who present themselves personally on the waiting list as part of routine management (eg, laboratory check for the reMELD-Na score) and fulfil the inclusion criteria should also be contacted personally.

### Randomisation

Patients are randomised as block randomisation in a 1:1 ratio. Randomisation is centrally managed through the electronic case report form (eCRF). This means that patients will be evenly distributed between the two study arms (control group with standard allocation and experimental group with the ExTra option).

To ensure an even distribution of key clinical characteristics across both treatment arms, the randomisation will be stratified according to the following prognostic variables:

- reMELD-Na-Score at the time of randomisation (<13 or ≥13),
- Waiting time prior to randomisation (0–6 months or >6 months).

### Interventions

#### Experimental arm

The experimental intervention, that is, the ExTra option, will be offered for a period of 12 months from randomisation, due to the expected medical deterioration of patients on the waiting list. In the experimental arm, treatment consists of either:

1. ExTra-LT: receiving a liver transplant with a graft that has been declined by all German transplant centres but meets established objective quality criteria during NMP.
2. LT: receiving a liver transplant via the regular liver allocation process when a graft becomes available, whichever occurs first.

Graft procurement and liver allocation are handled by Eurotransplant, in cooperation with the German organ procurement organisation DSO. To the best of our knowledge, organs are procured ethically and are not sourced from executed prisoners or prisoners of conscience or other vulnerable groups. The study team is not involved in the care of organ donors prior to organ donation. In Germany, post-mortem organ donation is regulated by the German Transplantation Act: the DSO coordinates the donation process on behalf of the donor hospitals, including donor identification and evaluation, verification of consent documentation and the logistical coordination of donor management, organ procurement and transport, whereas Eurotransplant is responsible for allocating the procured organs across its member countries according to defined, MELD-based allocation rules for liver grafts.

#### Quality assessment

Declined liver grafts considered for quality assessment must not have macrovesicular steatosis >60%, fibrosis grade >F3 or cirrhosis (based on histopathologic quick section analysis, if requested by the responsible transplant surgeon after macroscopic examination) and have a graft weight between 1 and 2.5 kg. Moreover, grafts must be connectable in terms of vascular anatomy. The goal is to maintain a dropout rate of no more than 50% for grafts that either fail quality assessment via NMP or cannot be safely transplanted despite undergoing NMP quality assessment. Additionally, the organ must not yet have been treated using a dynamic preservation procedure (HOPE or NMP).

As part of the ExTra LT quality assessment, the liver is removed from the cold preservation solution in the transplant centre in an end-ischaemic ‘back-to-base’ approach, prepared, cannulated and connected to the liver perfusion machine according to the respective manufacturer’s specifications. The decision to use or reject a graft after quality assessment by NMP (ie, start of recipient hepatectomy) is made at least 4 hours after the start of perfusion by the transplant surgeon, based on:

- Lactate levels <2.5 mmol/L.
- And two or more of the following:
  - Evidence of bile production.
  - Maintenance of a perfusate pH >7.30 (without correction for at least 1 hour).
  - Glucose metabolism (falling perfusate glucose value with consistent downward trend).
  - Maintenance of stable arterial and portal venous flows (if using OrganOx Metra: 150 and 500 mL/min, respectively).
  - Homogeneous perfusion with soft consistency of the parenchyma.

If the liver fulfils the above criteria after 4 hours of perfusion and is assessed as safely transplantable based on the assessment of the transplant surgeon in charge, the recipient is then initiated for surgery and the liver is transplanted. The quality assessment is device-independent (currently usable: XVIVO LiverAssist, OrganOx Metra or Aferetica PerLife). Additionally, a combination of HOPE and COR prior to the 4 hours of quality assessment with NMP is possible.

If patients are not transplanted within 12 months, they continue to be regularly listed on the waiting list without the possibility for an ExTra-LT and are censored.

#### Control arm

In the control arm, patients receive a liver graft following the regular allocation procedure. Both patients who receive an ExTra LT or a liver graft after standard allocation are followed up in hospital and after discharge at 3 months, 6 months and 12 months.

#### Criteria for discontinuing or modifying allocated interventions

Participation in the ExTra trial is voluntary, and participants have the right to withdraw from the study at any time, at their own request and without giving reasons. The following events can be considered as discontinuation criteria:

- Personal wish of the patient.
- Pregnancy.
- Serious complications.
- Significant protocol violations.
- Loss of contact, change of location or change of treating physician.

In addition, there are specific criteria that can lead to exclusion:

- Withdrawal of consent to accept livers with expanded donor criteria.
- Organ failure requiring combined organ listing.
- Disease progression while listed as non-transplantable: If a patient is deemed non-transplantable due to an infection or other event and the reMELD-Na is >21, patients are not directly excluded. The period of non-transplantable status is documented, and the reMELD-Na score reevaluated once the patient reaches a transplantable state. Only if the patient is listed as status as transplantable and the reMELD-Na remains >21 points, patients can no longer participate in the study. Survival data is censored up until this point.

#### Strategies to improve adherence to interventions

Patients are managed by the respective study centre as part of the waiting list management for LT. This includes, for example, regular blood samples for updating the reMELD-Na score to determine urgency. As part of the study, compliance is expected to be similar to the waiting list for LT. Ensuring compliance is the responsibility of the respective study centres or the transplant programme.

### Outcomes

#### Primary endpoint

The primary endpoint ‘time-to-transplant’ is defined as the time between randomisation and transplantation. Results will be evaluated on the intention-to-treat cohort, the hazards between experimental arm and control arm are compared using cause-specific Cox proportional hazards models.

#### Secondary endpoints

The secondary endpoints include, among others, competing events (death, disease progression, recovery) and are also evaluated on the intention-to-treat cohort. Again, the time from randomisation until the occurrence of the event is examined. For death (corresponds to waiting list mortality) and the composite of disease progression and recovery, cause-specific Cox proportional hazards models are calculated. The same variables as for the primary endpoint are used for adjustment (reMELD-Na score and waiting time), in addition to perfusion device used (OrganOx Metra, LiverAssist or Aferetica PerLife), study centre, age and sex. Furthermore, of particular interest are the number of patients still listed for transplantation after 12 months, the overall patient survival (from randomisation to death), the quality of life (EuroQol-5 Dimension, 5-Level questionnaire - EQ-5D-5L), the graft utilisation rate (used organs/offered organs for patients on the waiting list) as well as transplantation-associated costs.

#### Translational endpoints

Biomarkers in tissue, perfusate, bile and laboratory chemistry for outcome prediction (Early Allograft Dysfunction according to the criteria of Olthoff *et al*^14^ as well as 3-month graft survival) through NMP. This includes DNA and RNA analyses for the aforementioned purpose and future research (ie, the purpose of the analyses will be determined in the future). Samples will be stored at the central biobank (Central Biobank Charité/BIH).

### Assessments

#### Medical history

Baseline medical history will be obtained at time of inclusion to characterise the clinical status of all participants prior to transplantation. Demographic variables including age, height, weight, sex, blood group and Rhesus factor will be recorded. The indication for LT will be documented. Disease severity will be captured through the reMELD-Na score, documented together with the date of assessment. Medical history, including relevant comorbidities, will be collected.

#### Waitlist report

Comprehensive waitlist data will be collected during the 12-month intervention period to describe pretransplant organ offer dynamics and clinical prioritisation. For each recipient, the number of primary organ offers, and extended/rescue allocation offers will be documented, including offers accepted for the patient but not transplanted. All declined organs will be recorded together with the timing of the decline—before procurement, during procurement or on arrival at the transplant centre—and the documented reason. Periods during which a patient was temporarily not transplantable will be captured. These assessments provide a detailed representation of waiting time burden, offer utilisation, and centre-level decision pathways including transplant activity.

### EQ-5D-5L

Patient-reported outcomes will be assessed using the EQ-5D-5L instrument. The instrument evaluates five dimensions of health: mobility, self-care, usual activities, pain/discomfort and anxiety/depression. Each dimension is rated across five severity levels, yielding a five-digit health state profile. In addition, patients will rate their overall health status using the EQ Visual Analogue Scale (VAS), a validated 0–100 scale anchored by ‘worst imaginable’ and ‘best imaginable’ health. The EQ-5D-5L will be measured at time of inclusion, end of intervention/prior to LT, at discharge from hospital as well as 3, 6 and 12 months after LT (if applicable).

#### Bloodwork

Blood sampling will be performed according to routine clinical practice, avoiding additional venipuncture whenever possible. Timepoints are at time of inclusion, end of intervention/prior to LT, postoperative days 1 to 7 and day 10, at discharge from hospital as well as 3, 6 and 12 months after LT (if applicable). Laboratory analyses have been minimised to reduce participant burden while maintaining the parameters necessary for evaluating perioperative risk and early graft function. The panel includes complete blood count, sodium, liver enzymes (Aspartate Aminotransferase (AST), Alanine Aminotransferase, Gamma-Glutamyl Transferase, Alkaline Phosphatase), bilirubin, International Normalized Ratio, creatinine and urea.

#### Donor information

Detailed donor characteristics will be recorded to support evaluation of graft quality and donor-derived risk factors. Donor demographics, blood group, Rhesus factor, viral serologies (HCV antigen, HBc antibody, HIV antigen) and whether the graft was split will be documented. The location of donation and cause of death will be included. Donor management data, including vasopressor requirements and a complete biochemical profile, will be collected. Cold ischaemia time will be calculated from the initiation of cold perfusion in the donor to the time the graft is removed from ice at the transplant centre or when normothermic perfusion begins.

#### Preoperative status

The clinical condition immediately prior to transplantation will be documented, including the reMELD-Na score, the need for dialysis or mechanical ventilation and the administration of norepinephrine or other catecholamines. Time spent on the waiting list will be recorded in days.

#### Postoperative status

The early postoperative course will be evaluated through documentation of organ support requirements and transfusion needs. Data collected include the requirement for dialysis, mechanical ventilation, vasopressor therapy and the intraoperative administration of red blood cell concentrates, fresh frozen plasma, platelet concentrates, PPSB, tranexamic acid and fibrinogen. This stretches throughout the inpatient hospital stay of the LT and will be documented once in the eCRF at discharge.

#### Scores

##### Early Allograft Failure Simplified Estimation score

The Early Allograft Failure Simplified Estimation score will be determined on postoperative day 10 to quantify the risk of early allograft failure. The score integrates pretransplant disease severity, perioperative transfusion requirements, the occurrence of early postoperative thrombosis and the temporal evolution of AST, platelet count and bilirubin across the first 10 postoperative days. Centre transplant volume is incorporated as an additional parameter. The resulting composite metric offers a robust prediction of early graft failure.^28^

##### Balance of Risk Score

The Balance of Risk (BAR) score will be calculated prior to transplantation to estimate the risk of early graft failure. It incorporates the MELD score at transplant (excluding exception points), the need for retransplantation, pretransplant life support status, recipient age, cold ischaemia time and donor age. The BAR score supports risk stratification and provides prognostic context for early post-transplant outcomes.^15^

##### Comprehensive Complication Index

The Comprehensive Complication Index (CCI) will be used to quantify postoperative morbidity by integrating all postoperative complications into a single continuous score ranging from 0 (no complications) to 100 (death). Derived from the Clavien-Dindo classification but offering greater sensitivity and granularity, the CCI enables a comprehensive assessment of postoperative burden.^29 30^ The CCI will be calculated at hospital discharge and at postoperative day 90 to capture both early and later complications.

### Follow-up information

Follow-up evaluations will document all postoperative complications together with their diagnostic and therapeutic management. Additional clinical events—including rejection episodes, readmissions, retransplantation, relisting and death—will be recorded with corresponding dates and explanatory notes. Biliary complications will be documented in detail.

### MRCP

A routine Magnetic Resonance Cholangiopancreatography (MRCP) will be performed 12 months after transplantation to assess biliary anatomy and detect biliary complications. Examinations will be conducted at 1.5 T or 3 T using standardised sequences, including axial and coronal T2-weighted imaging, two-dimensional and three-dimensional MRCP, diffusion-weighted imaging and T1 in-phase/out-of-phase imaging. Gadolinium-based contrast administration is optional but recommended for vascular assessment, and hepatobiliary contrast agents may be used when clinically indicated. All costs for MRCP examinations are covered in the trial.

### Biobank sampling

**Figure 2:**
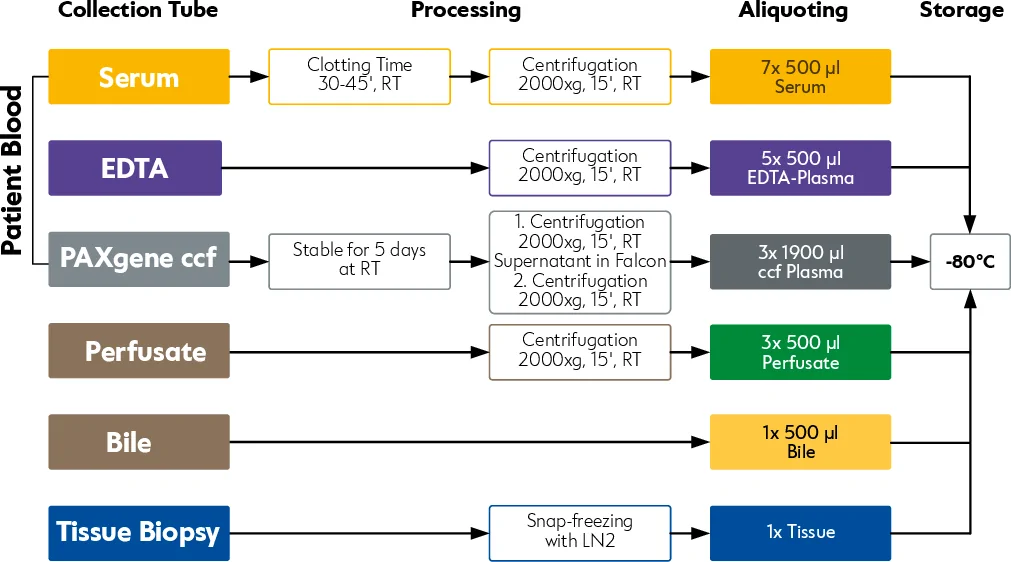
Biobank sample collection, preparation and storage in the ExTra trial. LN2, liquid nitrogen; RT, room temperature.

Participants will have the option to opt in for the biobank at the time of informed consent. Biobank procedures will be implemented uniformly across participating centres in collaboration with the Central Biobank Charité/BIH, which provides standardised collection kits, aliquoting materials and detailed processing instructions. Blood samples will be obtained at defined perioperative time points—before transplantation, 1 hour after reperfusion and on postoperative days 1 and 3—using routine clinical blood draws when possible (online supplemental table 2). These samples will include 10 mL of serum, 10 mL of EDTA whole blood, and one PAXgene blood sample. Since blood sampling is a routine part of preoperative care and postoperative follow-up, only additional material necessary for the study will be obtained. Liver wedge biopsies will be collected before machine perfusion or after organ preparation, after completion of NMP in the ExTra arm and 1 hour after reperfusion. Perfusate samples will be collected at defined time points, including before the start of perfusion, 15 min after initiation, at hourly intervals during the procedure and at the conclusion of NMP in the ExTra LT study arm, with 1.5 mL per sample. Additionally, bile samples will be collected every 2 hours during perfusion and at the end of NMP in the same study arm, with 500µl per sample. All samples will be processed locally (figure 2), frozen and transferred to the central biobank on dry ice, with transport costs covered by the project.

### Sample size

The primary statistical objective is to show that the cause-specific HR for the transplant risk between the two randomised groups is not equal to 1. The assumptions for the data-generating process are based on data from Eurotransplant in 2022: Within 12 months, 43.6% of patients with a MELD ≤25 (corresponds to reMELD-Na ≤21) who were not eligible for Standard or Non-Standard Exception were transplanted, 19.1% had a competing event (death, progression or recovery) and 37.2% remained on the waiting list and were censored in this study.

As a treatment effect, an increase in the transplantation rate of 20% to 63.6% was assumed, while the risk of the competing event remains unchanged. A 20% increase in the transplantation rate through the ExTra option is realistic, as 385 LTs were performed in Germany in 2022 in patients with MELD ≤25 without standard/non-standard exception MELD criteria, while 133 of 217 declined grafts would have been potentially acceptable after a quality assessment using NMP, representing approximately 20% additional grafts in the study population based on an estimated 50% acceptance rate following NMP.

Assuming exponential distributions for time to transplant and time to competing event, the data were simulated to fit this pattern. The parameter of the exponential distribution for time to transplant (in months) was 0.057 for the control group and 0.104 for the intervention group. The parameter for the competing risk was 0.0251 for both groups. A Cox proportional hazards model was used to estimate the transplant HR between the ExTra LT group and the control group.

Based on 1 000 000 simulations, a sample size of N=83 per group was calculated for a two-sided type I error of 0.05 and a power of 0.8. Considering a dropout rate of 10%, a total of 93 patients will be recruited per group. A median of 36 transplantations is expected in the control arm. It is expected that 4–29 additional transplantations (median: 17) will be performed in the experimental arm.

This number of cases can probably be reached with a recruitment period of 36 months. A 12-month intervention phase is planned, followed by a 12-month follow-up phase. The study will thus end when all 83 patients in each treatment group have completed the 12-month follow-up after randomisation/transplantation.

### Study sites

In Germany, 18 of 19 LT centres have expressed their interest in participating in the study. Currently, 11 centres are actively recruiting patients.

### Patient and public involvement

The study design was discussed with representatives of the patient advocacy groups ‘Lebertransplantierte Deutschland e.V.’ and ‘Bundesverband der Organtransplantierten e.V.’ which support all described measures to potentially increase the availability of life-saving LTs. Moreover, the trial concept was presented to the Standing Commission on Organ Transplantation of the German Medical Association (Ständige Kommission Organtransplantation der Bundesärztekammer), which is the relevant central organisation in the system of medical self-administration in Germany.

## Ethics and dissemination

### Ethical considerations

Since all interventions and all medical devices used in the study are CE-certified and are used in accordance with the intended purpose defined by the respective manufacturer, without any additional burdensome or invasive measures, the study qualifies as a clinical study under §47 (3) of the German Medical Devices Act (MPDG). The study is conducted in accordance with the Professional Code of Conduct for Physicians (*Berufsordnung der Ärzte*).

The study protocol (V1.0) received approval from the Institutional Review Board (IRB) of the Charité – Universitätsmedizin Berlin on 2 April 2025 (EA1/04/25). Subsequent amendments to the study protocol and patient informed consent form were approved on 3 June, 26 September and 27 November 2025 (protocol V1.2). Participating study centres will need local IRB approval prior to initiation (table 1). The detailed trial protocol is shared with participating study centres prior to initiation and interested parties on reasonable request. This study complies with Good Clinical Practice guidelines and all applicable national and local regulations. The trial has been registered at ClinicalTrials.gov (NCT06874296). The study started on 2 June 2025 and is expected to end on 31 December 2030.

**Table 1:** Enrolled study centres and their ethical approval status*

No.	Study site	Ethical approval
1	Charité – Universitätsmedizin Berlin	Yes
2	University Hospital RWTH Aachen	Yes
3	University Hospital Münster	Yes
4	University Medical Center Hamburg-Eppendorf	Yes
5	University Hospital Bonn	Yes
6	Eberhard Karls University of Tübingen	Yes
7	LMU University Hospital, LMU Munich	Yes
8	University Hospital Regensburg	Yes
9	University Hospital Würzburg	Yes
10	Hannover Medical School	Yes
11	University Hospital Essen	Yes
12	Heidelberg University Hospital	Yes
13	Jena University Hospital	Yes

* As of July 2026.

LMU, Ludwig-Maximilians-Universität München; RWTH, Rhenish-Westphalian Technical University Aachen.

### Data and safety monitoring

Monitoring follows a risk-based plan with regular on-site visits, verification of source data and review of laboratory values and adverse events. Investigators permit full access for monitoring, auditing and regulatory inspections, and Clinical Research Associates and auditors have direct access to eCRFs and source documents to ensure data accuracy and protocol compliance. Inconsistencies, implausible or missing data are flagged by a data management query in the eCRF. The potentially incorrect information can be corrected by directly changing the values in the eCRF or must be marked as accepted errors or false reports. The data transfer between the trial site and the study server is carried out via an encrypted connection so that the transferred study data cannot be manipulated. Patient data is only stored in pseudonymised form.

Safety oversight focuses on 3-month graft and patient survival (retransplantation or death), assessed using Kaplan-Meier analysis. Pseudonymised safety data are submitted quarterly by the principal investigator, and the independent Data Safety Monitoring Board (DSMB). The DSMB is composed of two hepatologists and two transplant surgeons with extensive expertise in liver graft assessment and NMP. They review the protocol, safety procedures and endpoints during the study in accordance with EMA guidance (EMEA/CHMP/EWP/5872/03 Corr) and may recommend early study termination for safety reasons.

The Steering Committee provides scientific oversight, meets regularly to monitor recruitment, study progress and protocol adherence and re-evaluates the benefit–risk profile based on the DSMB recommendations. It is responsible for decisions on study continuation or discontinuation, review of results, determination of presentation and publication strategies and the selection of secondary analyses and related publications.

### Dissemination plan

Primary study results will be published in peer-reviewed journals. All manuscripts, abstracts and presentations will undergo review by the Steering Committee to ensure scientific integrity, consistency and appropriate authorship. Data will be analysed and reported on a study-wide level; participating centres may not publish centre-specific results without prior approval. The principal investigator will coordinate the final report, which must be reviewed and approved by all centres before submission.

Secondary analyses or additional publications require Steering Committee approval, with proposing investigators leading development and authorship determined according to established guidelines. Study findings will also be disseminated through conferences, professional meetings, clinical trial registries and public engagement activities.

All investigators are bound to strict confidentiality until official publication, and no study data may be shared without authorisation from the principal investigator.

De-identified participant data, including the accompanying data dictionary, as well as the statistical analysis code and relevant study materials, will be made available on reasonable request to the principal investigator after publication of the primary results. Access will require submission of a brief research proposal outlining the planned use, which will be reviewed for scientific merit, ethical compliance and data-protection considerations.

## Supplementary material

10.1136/bmjopen-2025-114833 online supplemental file 1
