# Supplementary material for: Open, prospective, randomised, multicentre trial on quality assessment of declined liver grafts by normothermic ex situ machine perfusion for decreasing time to transplantation (ExTra trial): a study protocol
Source: BMJ Open. 2026 Aug 13;16(8):e114833. doi: 10.1136/bmjopen-2025-114833 (PMC13475461; doi:10.1136/bmjopen-2025-114833)
Supplement: Supplementary file 1 [file bmjopen-2025-114833.reviewer_comments.pdf]

PEER REVIEW HISTORY

BMJ Open publishes all reviews undertaken for accepted manuscripts. Reviewers are asked to complete a checklist review form and are provided with free text boxes to elaborate on their assessment. These free text comments are reproduced below.

ARTICLE DETAILS

Title (Provisional)

Open, prospective, randomized, multicentre trial on quality assessment of declined liver grafts by normothermic ex vivo machine perfusion for decreasing time to transplantation (ExTra trial): a study protocol

Authors

Moosburner, Simon; Gassner, Joseph M.G.V; Sauer, Igor M; Mandac, Mateja; Modest, Dominik P.; Zocholl, Dario; Stegherr, Regina; Patel, Madhukar S; Wendon, Julia; Roderburg, Christoph; Selzner, Markus; Pascher, Andreas; Fehrenbach , Uli; Pratschke, Johann; Raschzok, Nathanael

---

VERSION 1 - REVIEW

**Reviewer**	**1**
**Name**	**Mazariegos, George V**
**Affiliation**	**Children's Hospital of Pittsburgh of UPMC**
**Date**	**25-Jan-2026**
**COI**	**Medical Advisory Board, Bridge to Life**

Thank you for the opportunity to review the study protocol.The protocol, study design are clear. My only recommendation would be to consider apriori if there should be additional inclusion criteria for this back to base protocol such as a predefined cold ischemic time on arrival to the preliminary recipient hospital.

**Reviewer**	**2**
**Name**	**Thomson, David**
**Affiliation**	**University of Cape Town**
**Date**	**02-Feb-2026**
**COI**	**None**

The authors are conducting a well thought out study to improve access to deceased donor livers using normothermic machine perfusion.

It is a well written manuscript and minor comments are made below for consideration by authors and editors.

1) The abstract presents the study in the Introduction primarily as assessing the “safe transplantation of liver grafts that would otherwise be discarded.”

   However the primary study endpoint is time-to-transplant. Safety and improved access to transplant are clearly relevant outcomes the study aims to address and would consider reframing the introduction of the abstract to these broader aims.

2) Logistical considerations (capacity) are often a factor in resource intensive interventions.

   Primary endpoint is time-to-transplant, defined as the period between randomisation and transplantation. This would reflect access on an individual level. Other factors may influence time-to-transplant during the study.

   Are the authors including an assessment of centre specific liver transplant activity within the study time period to assist readers with interpreting the study relevance given that centre practices can vary dramatically between centres and over time.

3) “A centralized biobank will collect perfusate, tissue, and blood samples for biomarker research.”

   The detail on this research component included in this paper would benefit from being expanded and referenced in the methodology. Additional detail on the protocol for biobanking in Germany could be referenced along with the consent processes. The biobanking component is not relevant to the study but merits inclusion as it aligns with the aims of BMJ in publishing research methodologies to grow collaboration and inform researchers of existing work underway.

   With this in mind some additional information would be useful while acknowledging that the precise specifics of a methodology are not available yet for this part of the project. This could be in the form of supplemental appendices, the relevance of which are explained in the text.

4) “Safety will be monitored through quarterly reviews by a Data and Safety Monitoring”

   This is well discussed. The specific names of the Safety Monitoring Board are mentioned. I am unsure if this is standard practice as it is not something I have not come across before and could potentially allow for pressure to be applied onto these individuals. Defer to the BMJ Open journal editorial practice in this regard.

5) “Objective tools for assessing graft function are not routinely used in clinical practice for conventional static cold storage or hypothermic oxygenated liver machine.”

   This statement is quite categorical and the reference supplied in support of this statement doesn’t support it fully.

   Some objectivity is attempted to be applied in the real world. What counts as truly objective may be open to debate. Would the authors consider: “a lack of internationally validated criteria” or “lack of standardization” to be more appropriate ways to frame this statement?

6) “Able to consent”

Some additional detail on consent could be included.

Time points: Is consent at the time point of listing and then again at time of organ offer?
Is consent from a surrogate possible in cases of incapacity?
Are the patient information and consent forms able to be included as appendices?

Even if shared in the local languages of the study as appendices, as readers can use AI to translate if needed. Defer to BMJ Open editorial practices in this regard.

7) Lactate clearance < 2.5 mmol/L

   This term is expressed as a percentage in other medical studies. Could the authors clarify this term "clearance" and how it will be assessed in this study. Is this an end of NMP run lactate level of < 2.5 mmol/L. Or related to a clearance rate over time relative to starting lactate?

8) "The ExTra trial will randomize patients on the waiting list to demonstrate that declined liver grafts, which meet predefined quality criteria after at least four hours of NMP, can be used to ***safely*** reduce the waiting time for liver transplantation in patients with a low MELD score who are not eligible for Standard or Non-Standard Exemption."

   Consider whether "safely" should be introduced through the paper to highlight the dual aim of the study to assess time to transplant and the safety of these livers.

9) Given that the study context is key for international readers to assess impact in their own environment. Can the "not eligible for Standard or Non-Standard Exception criteria" be given to the reader in some form. Referenced and possibly included as an appendix.

10) Is portal and venous flow rate measurements a unique variable to the OrganOx Metra - or are other NMP devices able to measure this? The methodology links this variable to one specific device.

    "Maintenance of stable arterial and portal venous flows (OrganOx Metra: 150 and 500 mL/min respectively)"

    Can the authors clarify this.

11) The assessments section is comprehensive. Two comments in this section:

    a) The waitlist report - will the patients be cohorted into centres to assess centre influence - this may have been addressed in an earlier response

    b) The early postoperative course section - at what time point / periods will variables be measured during this period. This is not currently an easily reproducible component of the study from the methodology

12) Is the biobanking under a separate consent form or an inclusion within the consent form of this study? This may have been responded to under an earlier comment.

Overall a very well thought out and pragmatic study that will help advance our scientific understanding of the role of normothermic machine perfusion in expanding access to safe liver transplantation.

VERSION 1 - AUTHOR RESPONSE

Reviewer: 1

Dr. George V Mazariegos, Children's Hospital of Pittsburgh of UPMC

Comments to the Author:

Thank you for the opportunity to review the study protocol.The protocol, study design are clear. My only recommendation would be to consider apriori if there should be additional inclusion criteria for this back to base protocol such as a predefined cold ischemic time on arrival to the preliminary recipient hospital.

> *Dear Dr. Mazariegos, thank you for your expert assessment and recommendations. Indeed we did consider limiting previous cold ischemia time, however we feared potentially missing grafts if imposing a strict cut-off. From our experience this can highly vary and the logistics in the Eurotransplant region are not always predictable. The decision to accept a graft in the intervention groups remains with the transplant surgeon. However, we do have other strict cut-offs, which we have now added to the quality assessment section of the manuscript. These include steatosis >60% of the graft, fibrosis >FIII, graft weight and vascular anatomy.*

Reviewer: 2

Prof. David Thomson, University of Cape Town

Reviewer Comments

Pilot, open, prospective, randomized, multicentre trial on quality assessment of declined liver grafts by normothermic ex vivo machine perfusion for decreasing time to transplantation (ExTra trial): a study protocol. The authors are conducting a well thought out study to improve access to deceased donor livers using normothermic machine perfusion. It is a well written manuscript and minor comments are made below for consideration by authors and editors.

The abstract presents the study in the Introduction primarily as assessing the "safe transplantation of liver grafts that would otherwise be discarded."However the primary study endpoint is time-to-transplant. Safety and improved access to transplant are clearly relevant outcomes the study aims to address and would consider reframing the introduction of the abstract to these broader aims.

> *Dear Prof. Thomson thank you for your input. We have rephrased the introduction of the abstract to better reflect our primary endpoint: "The randomized multicentre ExTra trial aims to decrease time-to-transplant by safely improving access to transplantation through viability assessment of otherwise discarded grafts with normothermic machine perfusion (NMP)."*

Logistical considerations (capacity) are often a factor in resource intensive interventions. Primary endpoint is time-to-transplant, defined as the period between randomisation and transplantation. This would reflect access on an individual level. Other factors may influence time-to-transplant during the study. Are the authors including an assessment of centre specific liver transplant activity within the study time period to assist readers with interpreting the study relevance given that centre practices can vary dramatically between centres and over time.

We do agree with you, that centre-level transplant activity is of high relevance and will include this in our secondary analysis. This is now more clearly describe in the assessment section.

"A centralized biobank will collect perfusate, tissue, and blood samples for biomarker research." The detail on this research component included in this paper would benefit from being expanded and referenced in the methodology. Additional detail on the protocol for biobanking in Germany could be referenced along with the consent processes. The biobanking component is not relevant to the study but merits inclusion as it aligns with the aims of BMJ in publishing research methodologies to grow collaboration and inform researchers of existing work underway. With this in mind some additional information would be useful while acknowledging that the precise specifics of a methodology are not available yet for this part of the project. This could be in the form of supplemental appendices, the relevance of which are explained in the text.

Thank you for your advice. We have updated the biobank sampling section of our manuscript with supplementary table 2, which details the exact timepoints of collection and additionally added figure 2, explaining the processing of the samples. Consent will be obtained as an opt-in option at time of study inclusion.

"Safety will be monitored through quarterly reviews by a Data and Safety Monitoring". This is well discussed. The specific names of the Safety Monitoring Board are mentioned. I am unsure if this is standard practice as it is not something I have not come across before and could potentially allow for pressure to be applied onto these individuals. Defer to the BMJ Open journal editorial practice in this regard.

We have removed the names from the corresponding section of the manuscript.

"Objective tools for assessing graft function are not routinely used in clinical practice for conventional static cold storage or hypothermic oxygenated liver machine." This statement is quite categorical and the reference supplied in support of this statement doesn't support it fully. Some objectivity is attempted to be applied in the real world. What counts as truly objective may be open to debate. Would the authors consider: "a lack of internationally validated criteria" or "lack of standardization" to be more appropriate ways to frame this statement?

We agree, that this statement might be considered overly broad and does not reflect the work done by the Zurich group on FMN and NADH measurement. We have ammended this statement and furthermore reference the current ELITA guidelines, which do recommend further international validation.

"Able to consent"Some additional detail on consent could be included. Time points: Is consent at the time point of listing and then again at time of organ offer? Is consent from a surrogate possible in cases of incapacity? Are the patient information and consent forms able to be included as appendices? Even if shared in the local languages of the study as appendices, as readers can use AI to translate if needed. Defer to BMJ Open editorial practices in this regard.

The patient information and consent forms will available as supplementary material. Patients are consented once while on the waitlist. A consent from a surrogate is not intended. If the patient can give consent but is unable to sign personally, a witness must confirm the oral explanation by signing. We have updated the inclusion criteria section to better reflect this.

Lactate clearance < 2.5 mmol/L. This term is expressed as a percentage in other medical studies. Could the authors clarify this term "clearance" and how it will be assessed in this study. Is this an end of NMP run lactate level of < 2.5 mmol/L. Or related to a clearance rate

over time relative to starting lactate?

The term "clearance" in this context might have been misleading. We want perfusate lactate levels to be below 2.5 mmol/L after at least 4 hours of NMP. We have rephrased this section and hope this clarifies this criterion.

"The ExTra trial will randomize patients on the waiting list to demonstrate that declined liver grafts, which meet predefined quality criteria after at least four hours of NMP, can be used to safely reduce the waiting time for liver transplantation in patients with a low MELD score who are not eligible for Standard or Non-Standard Exemption." Consider whether "safely" should be introduced through the paper to highlight the dual aim of the study to assess time to transplant and the safety of these livers.

Thank you for this helpful comment. We agree, that this wording would strengthen our messaging and have applied this throughout the manuscript.

Given that the study context is key for international readers to assess impact in their own environment. Can the "not eligible for Standard or Non-Standard Exception criteria" be given to the reader in some form. Referenced and possibly included as an appendix.

We completely agree. We have explained SE/NSE points in the introduction and referenced the Eurotransplant Allocation Manual which details the relevant criteria.

Is portal and venous flow rate measurements a unique variable to the OrganOx Metra - or are other NMP devices able to measure this? The methodology links this variable to one specific device."Maintenance of stable arterial and portal venous flows (OrganOx Metra: 150 and 500 mL/min respectively)" Can the authors clarify this.

This methodology follows the protocol first published by Hynek Mergental 2016 (AJT; doi: 10.1111/ajt.13875) and only applies to the OrganOx Metra. Other NMP devices such as the XVIVO LiverAssist device allow the adjustment of the flow so that this criterion cannot apply. We have ammended the section to state "if using" to better clarify this.

The assessments section is comprehensive. Two comments in this section: The waitlist report - will the patients be cohorted into centres to assess centre influence - this may have been addressed in an earlier response

Yes this will be a secondary analysis. See waitlist report section.

The early postoperative course section - at what time point / periods will variables be measured during this period. This is not currently an easily reproducible component of the study from the methodology

We have improved the assessment section and have clarified the timepoints for analysis, including the early postoperative course. This analysis stretches throughout the in-patient hospital stay of the liver transplantation and will be documented once in the eCRF at discharge.

Is the biobanking under a separate consent form or an inclusion within the consent form of this study? This may have been responded to under an earlier comment.

No, patients will be consented at the same time. It is an optional opt-in choice for the patients. See section biobanking.

Overall a very well thought out and pragmatic study that will help advance our scientific understanding of the role of normothermic machine perfusion in expanding access to safe liver transplantation.

Dear Prof. Thomson thank you for your critical revision of our manuscript. We appreciate your input and do think this has improved our manuscript.

VERSION 2 - REVIEW

**Reviewer**	**2**
**Name**	**Thomson, David**
**Affiliation**	**University of Cape Town**
**Date**	**28-May-2026**
**COI**	

The authors have repsonded to all comments. The manuscript reads well and is scientifically sound and well thought out. There are 4 minor points on this read through for consideration by the authors where new additions to the text could be refined.

1)

Patients are included while are active on the waitlist.

Grammar - the “are” can be removed.

Could be rephrased as “Patients are eligible for enrollment while active on the waiting list”

2)

By piloting this procedure across participating study centres, we aim to safely transform organ acceptance practices in Germany, provided the results are successful.

“Results are successful” is always a term open to interpretation. And there are multiple results within this study possible. Would be specific around which results the authors feel would result in a change in acceptance practice.

Could be rephrased as “By piloting this procedure across participating study centres and proving increased access and acceptable patient outcomes, we aim to safely transform organ acceptance practices in Germany.”

3)

How does ***“The duration of waitlist time before study inclusion is included in the randomisation stratification.”*** get factored into stratification? Would need additional explanation.

4)

The primary recruitment measure should be a personal discussion as part of the information on liver transplantation during consultation hours. For this purpose, the respective doctors providing information and the transplant office in the recruiting

centres will be informed and sensitized. In addition, information flyers will be made available in the respective transplant outpatient clinics so that potential study participants can inform themselves independently. All patients who present themselves personally on the waiting list as part of routine management (e.g. laboratory check for the ReMELD-Na score) and fulfil the inclusion criteria should also be contacted personally.

Some grammatical suggestions to the phrasing.

“Measure” could be “process”
“Should be” is made more clear as “will be”
“personal “ is intended as “in-person”
“Should also” is made more clear with “will also”

VERSION 2 - AUTHOR RESPONSE

Reviewer: 2
Prof. David Thomson, University of Cape Town
Reviewer Comments

The authors have responded to all comments. The manuscript reads well and is scientifically sound and well thought out. There are 4 minor points on this read through for consideration by the authors where new additions to the text could be refined.

Dear Prof. Thomson, thank you very much for your feedback!
