# Supplementary material for: Open, prospective, randomised, multicentre trial on quality assessment of declined liver grafts by normothermic ex situ machine perfusion for decreasing time to transplantation (ExTra trial): a study protocol
Source: BMJ Open. 2026 Aug 13;16(8):e114833. doi: 10.1136/bmjopen-2025-114833 (PMC13475461; doi:10.1136/bmjopen-2025-114833)
Supplement: Supplementary file 2 [file bmjopen-16-8-s001.docx]

***Supplementary Material:***

Open, prospective, randomized, multicentre trial on quality assessment of declined liver grafts by normothermic ex vivo machine perfusion for decreasing time to transplantation (ExTra trial): a study protocol

**Supplementary table 1** Schedule of enrolment, interventions and assessments

|  | **TRIAL PERIOD** | | | | | | | | |
| --- | --- | --- | --- | --- | --- | --- | --- | --- | --- |
|  | Enrolment | | Intervention Period | | NMP | LT^d^ | Post-transplant follow-up | | |
| **TIMEPOINT** | -t_i_ to 0 | 0 | 0M^a^ | 12M^a^ | t_x_ | t_x+y_ | 3M^a,b^ | 6M^a,b^ | 12M^a,b^ |
| **ENROLLMENT** |  |  |  |  |  |  |  |  |  |
| Eligibility screen | X |  |  |  |  |  |  |  |  |
| Informed consent | X |  |  |  |  |  |  |  |  |
| Bloodwork (ReMELD-Na) | X |  |  |  |  |  |  |  |  |
| Randomisation |  | X |  |  |  |  |  |  |  |
| **INTERVENTION/ COMPARATOR** |  |  |  |  |  |  |  |  |  |
| ExTra option |  | X |  |  | X | X | X | X | X |
| Standard of Care |  | X |  |  | (X)^c^ | X | X | X | X |
| **ASSESSMENTS** |  |  |  |  |  |  |  |  |  |
| Medical History |  | X |  |  |  |  |  |  |  |
| Waitlist Report |  |  |  | X |  |  |  |  |  |
| EQ5DL |  | X |  | X |  | X | X | X | X |
| Bloodwork |  |  |  | X |  | X | X | X | X |
| Donor Information |  |  |  |  |  | X |  |  |  |
| Preoperative Status |  |  |  |  |  | X |  |  |  |
| Quality Assessment |  |  |  |  | X |  |  |  |  |
| Postoperative Status |  |  |  |  |  | X |  |  |  |
| Scores (BAR, EASE, CCI) |  |  |  |  |  | X |  |  |  |
| Follow-up Information |  |  |  |  |  |  | X | X | X |
| MRCP |  |  |  |  |  |  |  |  | X |
| Biobank Sampling |  |  |  |  | X | X |  |  |  |

^a^months

^b^acceptable time window ± 2 weeks

^c^optional, no quality assessment in control group

^d^in hospital assessments on postoperative days 1-10 (bloodwork, EQ5DL on discharge)

**Supplementary table 2** Biobank sampling timepoints

|  |  |  | **NMP only** | | | | | |  |  |  |
| --- | --- | --- | --- | --- | --- | --- | --- | --- | --- | --- | --- |
| **TIMEPOINT** | Before Transplantation | After Backtable | 15 min. NMP | 2h NMP | 4h NMP | 6h NMP | 8h NMP | 10h NMP/End of NMP | 1h post reperfusion | Postoperative day 1 | Postoperative day 3 |
| Patient blood | X |  |  |  |  |  |  |  | X | X | X |
| Liver tissue biopsy |  | X |  |  |  |  |  |  | X |  |  |
| Perfusate |  |  | X | X | X | X | X | X |  |  |  |
| Bile |  |  | X | X | X | X | X | X |  |  |  |

Abbreviations: Normothermic Machine Perfusion (NMP), hours (h), minutes (min)
